# Robotic Replica of a Human Spine Uses Soft Magnetic Sensor Array to Forecast Intervertebral Loads and Posture after Surgery

**DOI:** 10.3390/s22010212

**Published:** 2021-12-29

**Authors:** Maohua Lin, Moaed A. Abd, Alex Taing, Chi-Tay Tsai, Frank D. Vrionis, Erik D. Engeberg

**Affiliations:** 1 Department of Ocean and Mechanical Engineering, Florida Atlantic University, Boca Raton, FL 33431, USA; mlin2014@fau.edu (M.L.); mabd2015@fau.edu (M.A.A.); tsaict@fau.edu (C.-T.T.); 2 Department of Biomedical Engineering, University of Virginia, Charlottesville, VA 22904, USA; alt6yzw@virginia.edu; 3 Department of Neurosurgery, Marcus Neuroscience Institute, Boca Raton Regional Hospital, Boca Raton, FL 33486, USA; 4 Center for Complex Systems and Brain Sciences, Florida Atlantic University, Boca Raton, FL 33431, USA

**Keywords:** soft magnet, sensor array, machine learning, 3D printing, cervical spine, artificial disc

## Abstract

Cervical disc implants are conventional surgical treatments for patients with degenerative disc disease, such as cervical myelopathy and radiculopathy. However, the surgeon still must determine the candidacy of cervical disc implants mainly from the findings of diagnostic imaging studies, which can sometimes lead to complications and implant failure. To help address these problems, a new approach was developed to enable surgeons to preview the post-operative effects of an artificial disc implant in a patient-specific fashion prior to surgery. To that end, a robotic replica of a person’s spine was 3D printed, modified to include an artificial disc implant, and outfitted with a soft magnetic sensor array. The aims of this study are threefold: first, to evaluate the potential of a soft magnetic sensor array to detect the location and amplitude of applied loads; second, to use the soft magnetic sensor array in a 3D printed human spine replica to distinguish between five different robotically actuated postures; and third, to compare the efficacy of four different machine learning algorithms to classify the loads, amplitudes, and postures obtained from the first and second aims. Benchtop experiments showed that the soft magnetic sensor array was capable of precisely detecting the location and amplitude of forces, which were successfully classified by four different machine learning algorithms that were compared for their capabilities: Support Vector Machine (SVM), K-Nearest Neighbor (KNN), Random Forest (RF), and Artificial Neural Network (ANN). In particular, the RF and ANN algorithms were able to classify locations of loads applied 3.25 mm apart with 98.39% ± 1.50% and 98.05% ± 1.56% accuracies, respectively. Furthermore, the ANN had an accuracy of 94.46% ± 2.84% to classify the location that a 10 g load was applied. The artificial disc-implanted spine replica was subjected to flexion and extension by a robotic arm. Five different postures of the spine were successfully classified with 100% ± 0.0% accuracy with the ANN using the soft magnetic sensor array. All results indicated that the magnetic sensor array has promising potential to generate data prior to invasive surgeries that could be utilized to preoperatively assess the suitability of a particular intervention for specific patients and to potentially assist the postoperative care of people with cervical disc implants.

## 1. Introduction

Cervical spine degenerative disc disease can result in spinal cord injury and nerve root compression, leading to painful symptoms such as myelopathy and radiculopathy [1]. A cervical artificial disc implant is a conventional surgical treatment for these patients; however, the mechanical failure of these devices remains a significant complication, especially in patients with multilevel pathology [2,3]. Static disc pressure has been measured using a very small needle-type pressure sensor, but there are few methods to measure intervertebral pressure distribution [4,5]. As a result, the surgeon is forced to determine candidacy for cervical disc implants primarily on the findings of diagnostic imaging studies.

As an alternative, the use of wearable sensors in the acquisition of spinal motion parameters has shown promise throughout the literature [6,7]. Their quick and effortless integration makes them a promising technology that may become a supplement to high-cost imaging systems [8,9,10]. Accurate representation of the geometry and kinematics of a patient’s spine is the beginning of realistic phantoms and models [11,12]. Measurements of intradiscal pressure could be gained as well in the testing of the physical models. Literature reports several methodologies for creating sensor arrays, which can measure the pressure at several points across a surface [13,14]. However, flexible sensing technologies are required to integrate them into a spine model without inhibiting mobility.

Flexible magnetoelectronics have attracted many researchers in the past few years with different types of soft, compliant magnets [15]. A flexible magnetic sensor array is a new method to realize soft and stretchable magnets by mixing silicone with magnetic powder [16]. These sensors are low-cost, highly sensitive, and easily integrated into robotic systems as the soft medium can be manipulated in many shapes and sizes [16,17]. Recently, Hall effect sensors have been used in conjunction with a wearable magnetic skin for contact location and force measurement [15,16,17].

**Figure 1:**
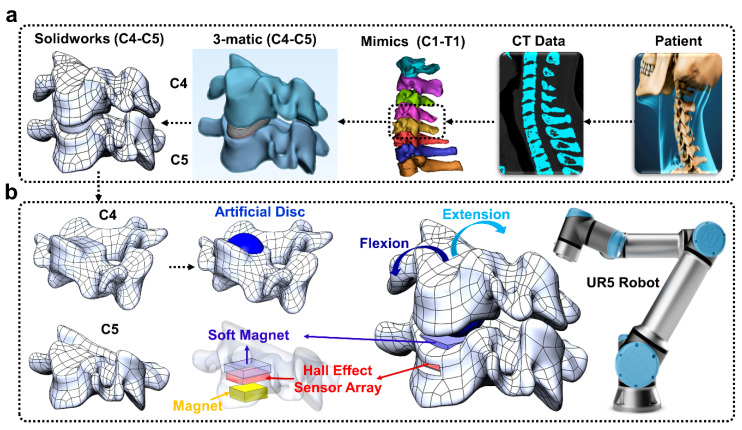
(**a**) Patient-specific robotic spine model based on a CT scan of the spine. (**b**) A modified artificial disc was ‘implanted’ into the C4 cervical spine replica. The soft magnet, Hall effect sensor array board, permanent magnet, and Ecoflex were embedded in the C5 vertebra replica. A robotic arm flexed and extended the cervical spine replica while the intervertebral loads were monitored with the soft magnetic sensor array to classify the spine posture with four different machine learning algorithms.

The overall goal of this paper was to develop a new approach to enable surgeons to preview the post-operative effects of an artificial disc implant in a patient-specific fashion prior to surgery (Figure 1).

## 2. Materials and Methods

**Figure 2:**
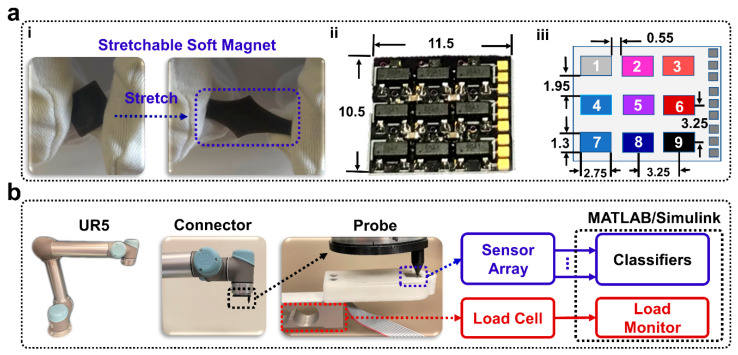
Robotically probing soft magnet to classify location and amplitude of external loads. (**ai**) The stretchable soft magnet. (**aii**) The Hall effect sensor array PCB with dimensional units of mm. (**aiii**) Each individual Hall effect sensor taxel position was numbered and physical dimensions were labeled with units of mm. (**b**) The UR5 applied loads to the soft magnet that was placed atop the 3 × 3 Hall effect sensor array within the 3D-printed housing. These nine Hall effect sensor signals were recorded in Simulink and classified with four machine learning algorithms in MATLAB.

The soft magnet was fabricated and placed atop a 3 × 3 array of Hall effect sensors. The soft sensing magnet comprised magnetic ferrofluid (Apex) mixed with a soft, stretchable elastomer (Figure 2(ai)). Deformation of the soft magnet by external loads displaced the magnetic particles within the soft magnet, and the resulting changes in the magnetic field were detected by the Hall effect sensor array (Figure 2(aii,aiii)). Four machine learning algorithms were compared to classify the amplitude and the locations that external loads were applied based on the sensor values reported by the nine Hall effect sensors on the 3 × 3 grid during robotic probing experiments (Figure 2b). The soft magnetic sensor array was next integrated into the artificial disc-implanted robotic replica of the human spine (Figure 1a) that was flexed and extended by a robotic arm (Figure 1b). Four different machine learning algorithms were next compared for their capabilities to classify five different postures of the human spine robotic replica.

### 2.1. Soft Magnet Fabrication

The soft magnet was realized by mixing Ecoflex 00-50 (Smooth-On) and ferrofluid magnetic liquid. Two variations of the soft magnetic sensor array were developed and compared by altering the ratios of Ecoflex and ferrofluid with the following weight ratios (Ecoflex part A–Ecoflex part B–ferrofluid): 1:1:0.2 (10% ferrofluid), 1:1:0.3 (15% ferrofluid). These ratios were mixed, degassed, poured into square 3D printed molds, covered with the mold cap, and allowed to cure for at least 12 h.

### 2.2. Robotically Probing Soft Magnets to Classify Location and Amplitude

The soft magnet, Hall effect sensor array, and permanent magnet were assembled within a 3D printed sensor housing (Figure 2a) and screwed securely to the end of a 2 kg load cell (LSP-2, Transducer Techniques, Temecula, CA, USA) that was clamped onto a desktop (Figure 2b). A 10 mm × 10 mm square permanent magnet was located 3 mm below the Hall effect sensor array PCB to strengthen the impact of displacing the magnetic particles within the soft magnet near the Hall effect sensors. The Hall effect sensor array PCB was designed in Eagle and fabricated by OSH Park (Lake Oswego, OR, USA). Nine Hall effect sensors (DRV5055A1QDBZR with the A1 model corresponding to the sensitivity option of 100 mV/mT and a sensing range of ±21 mT; Texas Instruments, Dallas, TX, USA) were next soldered to the PCB (Figure 2(aii)). The Hall effect sensor array and the load cell were interfaced with a computer via a 16-bit PCIe-6323 data acquisition (DAQ) card (National Instruments, Austin, TX, USA). Data were sampled with a ±10 V range producing a resolution of 305 µV. To amplify the response of the load cell, an INA131 (Texas Instruments) instrumentation amplifier was used.

A UR5 robotic arm (Universal Robots, Odense, Denmark) was bolted onto a metal table adjacent to the soft magnetic sensor array. A probe was 3D printed using Ultimaker 3 (Ultimaker, Zaltbommel, the Netherlands) and bolted to the end effector of the UR5 arm (Figure 2b). The probe had a flat tip with a 4 mm^2^ surface area. Simulink (The MathWorks, Natick, MA, USA) was used as the data collection software for both the load cell and the Hall effect sensor array. Data were low pass filtered and sampled at 1 kHz.

The UR5 was programmed using Universal Robot’s proprietary user interface, PolyScope. This interface allowed programming the robot through manual control of the robotic arm to set waypoints, directing its movement through a sequence of target positions and loads, as measured by the load cell. The robot was programmed to probe the sensor in 9 different locations in a 3 × 3 grid; each probe location was directly above a different Hall effect sensor (Figure 2(aiii)). Thirty repetitions at each of the nine taxels (tactile pixel) were done with six different loads: 5 g, 10 g, 20 g, 50 g, 75 g, and 100 g. This procedure was replicated for both soft magnets that were comprised of 10% or 15% ferrofluid. This produced 3240 datasets (9 locations × 30 repetitions × 6 loads × 2 soft magnets) that were used to train four different machine learning algorithms which were compared for load amplitude and location detection accuracy with each soft magnet. The correlation between load and Hall effect sensor readings was also plotted with a linear fit model at each taxel location in MATLAB. From this, the sensitivity in mV/g was reported along with the R^2^ values to quantify the goodness of fit of the linear models.

To evaluate the measurement uncertainty of the soft magnetic sensors, we followed the guidelines from the National Institute of Standards and Technology (NIST) [18]. In our application, the main sources of measurement uncertainty in the force sensing application were caused by deviations of the measured force from the value predicted by the linear models for the external LSP-2 load cell ($U_{Load\;Cell}$) and also each taxel on the soft magnetic sensor array ($U_{Taxel,i}$). To calculate the uncertainty (U) for both of these sources, we used the same procedure outlined by NIST based on the difference between the measured values and the values predicted by the models fit to the data:

(1) $$U^{2}=\frac{\sum_{j=1}^{n}d_{j}^{2}}{\left(n-m\right)}$$

where $d_{j}$ is the difference between the measured response and the calculated response using the linear models fit to the data, *n* is the number of measurement repetitions and m is the order of the polynomial modeling the data plus one (*m* = 2). We fit linear models to data from the load cell and each taxel on the sensor array for both soft magnets. The number of repetitions for each of the nine taxels and the load cell was *n* = 30. We next calculated the overall measurement uncertainty for each taxel *i* of the sensor array:

(2) $$U_{i}{}^{2}=U_{Taxel,i}{}^{2}+U_{Load\;Cell}{}^{2}$$

$U_{Taxel,i\;}$ is the measurement uncertainty of each taxel *i* on the sensor array and $U_{Load\;Cell}$ is the uncertainty of the load cell, both of which were calculated using (1). $U_{i}\;$ is the overall measurement uncertainty for each taxel i (Figure 2(aiii)), including the load cell measurement uncertainty.

### 2.3. Robotic Replica of Human Spine to Preview Artificial Disc Implantation

The CT scan of a person’s cervical spine was imported into Solidworks™ (Waltham, MA, USA) to produce a CAD model (C4–C5 cervical spine, Figure 1a) using Mimics/3-matic [19,20] and Hypermesh [21,22,23]. The C4 vertebra model was virtually “implanted” with an artificial disc (modified from the ProDisc-C [24]) to preview the effects of the intervention prior to surgery (Figure 1b). The C4 vertebra model was also altered to allow a mechanical connection to the UR5 robotic arm end effector. This concept has a powerful potential to enable surgeons to preview and compare the effects of different surgical interventions in a patient-specific manner using robotically actuated spine replica. Similarly, the C5 vertebra was modified to house the soft magnet, Hall effect sensor array board, and permanent magnet. The C5 vertebra replica was also designed so that it could be bolted to an LSP-35 load cell (Transducer Techniques, Temecula, CA, USA) for load monitoring (Figure 1b). The C4 and C5 vertebrae were subsequently 3D printed with Ultimaker 3.

The artificial disc ‘implanted’ C4 replica was bolted to the UR5 arm, which was programmed to flex and extend the C4 vertebra relative to the C5 vertebra (30 times for each soft magnet). The soft magnetic sensor array was used to classify five different postures of the spine replica: center, mid-flexion, flexion, mid-extension, extension.

### 2.4. Machine Learning Classification Approach

Extracting features from time-domain data is helpful to train a machine learning classifier to recognize the patterns hidden within the data. In some cases, frequency features are extracted to help classify patterns within the data [25]. Another way to produce features is through statistical information to represent the data. In this paper, 15 statistical features were extracted from each dataset (Table 1). After extracting the statistical features from the time domain data, the feature matrices were used to train and compare four distinct machine learning algorithms: Support Vector Machine (SVM), K-Nearest Neighbor (KNN), Random Forest (RF), and Artificial Neural Network (ANN).

The SVM algorithm creates a separating hyperplane in the feature space between all the classes [26]. The SVM algorithm uses the extracted features for the training portion to generate the hyperplane. The maximum separation margin between the classes is calculated and the hyperplane is constructed in the middle of the margin. The KNN algorithm calculates the shortest distance between a query and classifies the new samples based on the majority vote of its neighbors. The classes are assigned based on the most common vote amongst its K nearest neighbor measured by the shortest distance. The points in the extracted features select the specified K number closest to the query to vote for the most repeated class number [27]. The RF algorithm contains a collection of tree predictors such that each tree is initialized with random values independently from each other with the same distribution of all trees in the forest [28]. As the number of trees in the forest increase, the generalization error approaches the minimum limit to ensure the best performance in classification and prediction. The RF is a very effective machine learning tool since it contains multiple trees—each tree is an independent classifier, and its classification accuracy is independent of other trees in the forest. The RF algorithm used in this study was designed with 500 trees to perform the classification assignment. In general, a forest with more trees produces more robust predictions which lead to higher classification accuracy. The neural network toolbox in MATLAB (nprtool) was used to generate a feedforward network to train and test the ANN [29]. A three-layer feedforward network with 100 hidden neurons with a sigmoid activation function for the hidden layer and output neurons with a softmax activation function for the output layer was used to classify the collected data. The extracted statistical features were fed into the ANN through the input layer which consists of the input neurons. The neurons in the output layer represent the output classes. The performance of the network was evaluated using cross-entropy and confusion matrices, and the network was trained with scaled conjugate gradient backpropagation.

In the robotic probing experiments, the collected data were used to train and test the machine learning classifiers in two different configurations. In the first configuration, the machine learning classifiers were used to distinguish between 10 different classes (9 taxel locations and no-touch), and in the second configuration, the machine learning classifiers were used to distinguish between 6 different loads (5 g, 10 g, 20 g, 50 g, 75 g, 100 g). To evaluate the sensor for the artificial disc-implanted spine replica application (Figure 1b), five output classes were defined for each classifier corresponding to five different postures of the spine that were robotically actuated: center, mid-flexion, flexion, mid-extension, and extension.

To train and test the ANN, the feature extracted dataset was separated into 3 groups: 70% training dataset, 15% validation dataset, and 15% testing dataset. The ANN was trained, and the network parameters were modified according to the error generated with the training dataset. The validation dataset was used to measure network generalization error and to stop training when the generalization error stopped improving, while the testing dataset was used to provide an independent evaluation of network performance after the training, and it is independent of the validation dataset. The training process automatically stopped when generalization error stopped improving, as indicated by an increase in the cross-entropy error of the validation dataset. For the SVM, KNN, and RF machine learning algorithms, the extracted features were subdivided randomly into two categories, the training data which included 80% of the feature data, and the testing data which included the remaining 20% of the extracted feature dataset. Overfitting can cause the generalized performance of any classification model to decrease significantly. To avoid overfitting problems, feature data were swapped randomly before training and testing the classifier models. To avoid any biases, each classifier model was run 10 times with randomized selection of the 80% training features and 20% testing features and the average and standard deviation of the classification accuracy was reported for each of the four algorithms.

### 2.5. Statistical Analysis

Two different three-factor analysis of variance (ANOVA) tests were performed to assess the statistical significance of the results from the two different configurations of machine learning algorithms used with the robotic probing experiments. In each case, the dependent variable was the classification accuracy of the machine learning algorithms. The independent variables for the first configuration were the taxel location where loads were applied (9 taxels), the soft magnet composition (10% or 15% ferrofluid), and the machine learning algorithm (ANN, KNN, SVM, or RF). The independent variables for the second configuration were the load amplitude (6 loads), the soft magnetic skin composition (10% or 15% ferrofluid), and the machine learning algorithm (ANN, KNN, SVM or RF).

A two-factor ANOVA was performed on the robotically actuated spine replica experiments. The independent variables were the soft magnet composition (10% or 15% ferrofluid) and the four different machine learning algorithms. The dependent variable was the accuracy to classify the five different postures of the spine. A *p*-value < 0.01 was used in all cases for statistical significance.

## 3. Results

**Figure 3:**
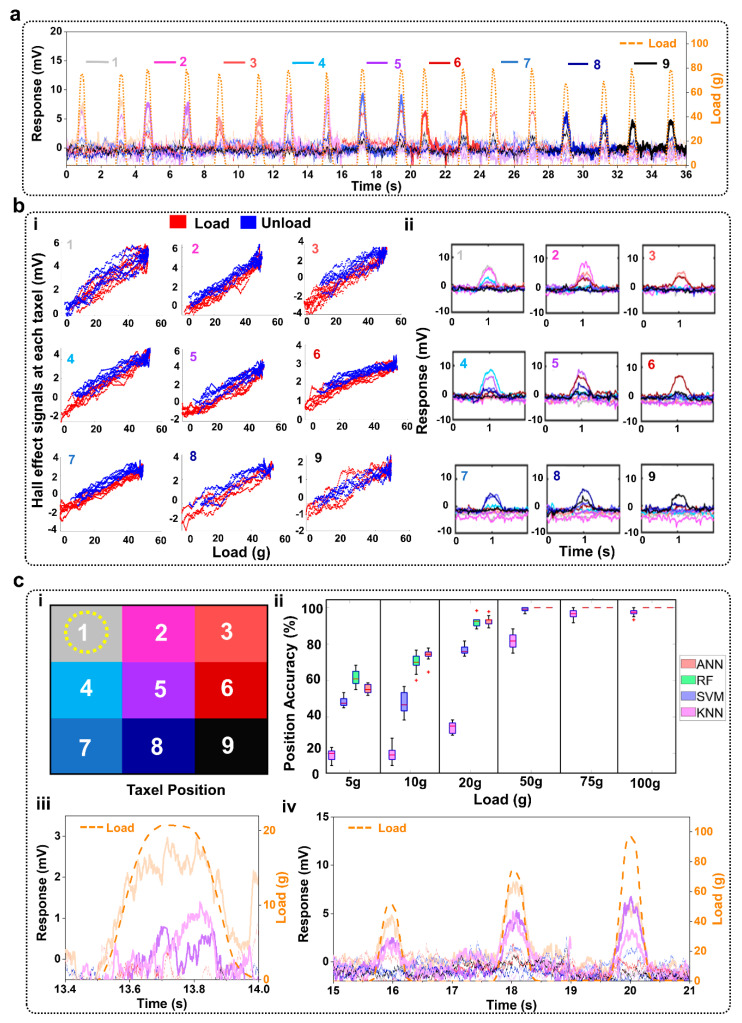
Classification accuracy of 10% ferrofluid soft magnetic sensor array. (**a**) Illustrative responses and load cell data at the nine taxel locations with a 75 g load. (**bi**) Load changes with Hall effect signals at each taxel, (**bii**) Individual taxel responses under 100 g load. (**ci**) Hall effect sensor taxel position. (**cii**) Classification accuracies to detect the different locations a load was applied with each of the six different loads (*n* = 30 repetitions/load). (**ciii**) Taxel responses under 20 g load applied at taxel 1. (**civ**) All taxel responses under different loads (50 g, 75 g, 100 g) applied at taxel 1.

**Figure 4:**
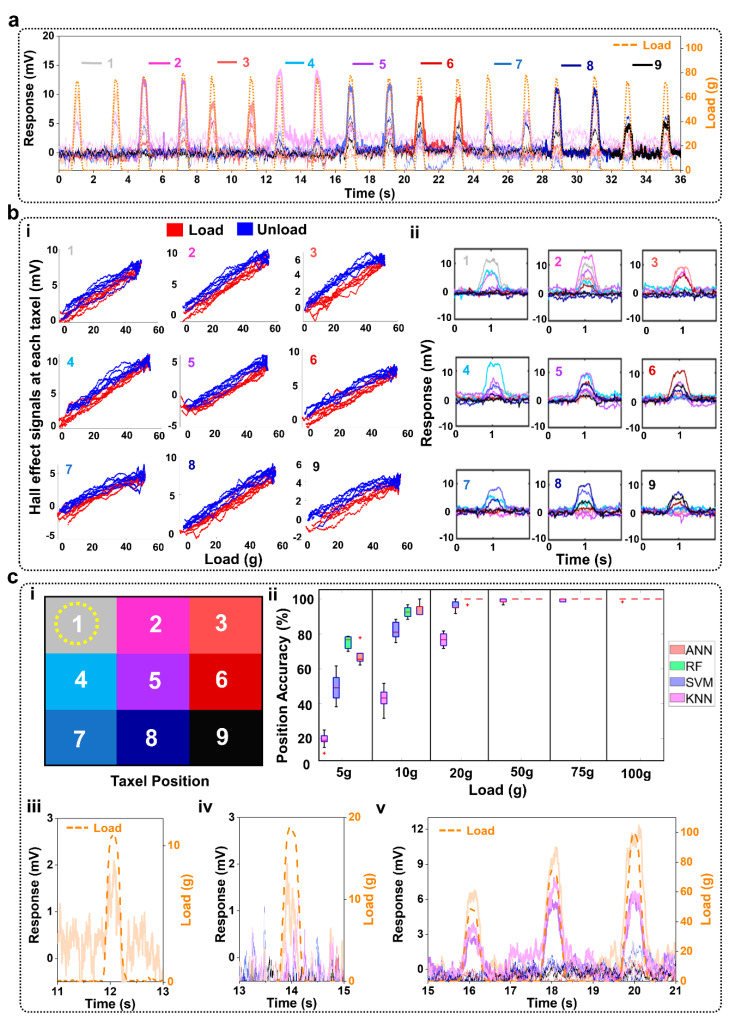
Classification accuracy of 15% ferrofluid Hall effect sensor array. (**a**) Illustrative responses and load data at the nine taxel locations. (**bi**) Load changes with Hall effect signals at each taxel, (**bii**) Individual taxel responses under 100 g load. (**ci**) Taxel location color code indicating illustrative data is from taxel 1. (**cii**) Classification accuracies to detect the different locations a load was applied with each of the six different loads (*n* = 30 repetitions/load). (**ciii**) Taxel 1 response to the 10 g load applied at taxel 1. (**civ**) All taxel responses from a 20 g load applied at taxel 1. (**cv**) All taxel responses under different loads (50 g, 75 g, 100 g) applied at taxel 1.

Sample time-domain data from the soft magnetic sensor array with the 10% ferrofluid composition showed the taxels’ responses as a 75 g load was applied repeatedly in nine different locations (Figure 3a). Spatially organized responses of each taxel showed similar trends of signals responding to different probing locations with the 100 g load (Figure 3(bii)). Corresponding time response data with a 75 g load (Figure 4a) and spatially organized responses with the 100 g load (Figure 4(bii)) show similar trends with the 15% ferrofluid soft magnet composition. The correlation between load and voltage measured by each of the nine Hall effect sensors for both soft magnets is reported in Figure 3(bi) and Figure 4(bi). A linear model was fit to these data. Averaged across all nine taxels, the R^2^ value of the linear fit for the 10% ferrofluid soft magnet was 0.88 ± 0.03 and the R^2^ value for the 15% ferrofluid soft magnet was 0.90 ± 0.04 (Table 2). Averaged across all nine taxels, the sensitivity for the 10% ferrofluid soft magnet was 0.09 mV/g ± 0.02 mV/g (Figure 3(bi), Table 2). The mean sensitivity of the 15% ferrofluid soft magnet was 0.14 mV/g ± 0.03 mV/g (Figure 4(bi), Table 2). The measurement uncertainties for the 9 taxels that were calculated according to (2) ranged between 3.23 g and 6.24 g for the 15% ferrofluid Soft Magnet (Table 3). Uncertainties for the 10% ferrofluid soft magnet followed similar trends with moderately higher levels of measurement uncertainty.

### 3.1. Classifying Locations of Applied Loads

At each location, the applied load triggered distinct patterns of taxel responses that were classified by the four different machine learning algorithms to distinguish between the probing locations for given loads. These algorithms showed high classification accuracies with both the 10% ferrofluid (Figure 3(cii)) and 15% ferrofluid soft magnets (Figure 4(cii)). In particular, the RF and ANN accuracies were respectively 98.39% ± 1.50% and 98.05% ± 1.56% with the 15% ferrofluid soft magnet (Figure 4(cii)).

Three-factor ANOVA showed that the classification accuracies were significantly impacted by the four different algorithms (*p* < 0.01), the two soft magnets (*p* < 0.01), and the nine different taxel locations (*p* < 0.01). The interaction between the classification algorithms and the load amplitudes was significant (*p* < 0.01). One illustration of this interaction can be seen with the higher accuracy of the RF compared to the ANN at the 5 g load whereas the ANN had a higher accuracy than the RF with the 10 g load. The interaction between the classification algorithms and the two different soft magnets was also significant (*p* < 0.01). This interaction can be seen, for example, with the accuracy of the KNN with 10% ferrofluid soft magnet (Figure 3(cii)) being significantly lower than the RF with 15% ferrofluid soft magnet (Figure 4(cii)). The load amplitudes and the ferrofluid percentage of the soft magnets also significantly interacted (*p* < 0.01). This interaction can be seen, in one illustrative case, as the accuracies for the 5 g loads with the 10% ferrofluid soft magnet (Figure 3(cii)) were significantly lower than the classification accuracies with the 10 g load and the 15% ferrofluid soft magnet (Figure 4(cii)). The three-factor interaction is significantly different (*p* < 0.01) because of the many cases where the classification accuracies were significantly different, for example, the SVM with 5 g load and the 10% ferrofluid soft magnet compared to the RF with the 10 g and 15% ferrofluid soft magnet.

### 3.2. Distinguishing between Different Loads at Each Taxel Location

The machine learning algorithms were able to distinguish between all the loads applied to each location with high classification accuracy for loads ≥ 20 g with the 10% ferrofluid soft magnet (Figure S1a) and loads ≥ 10 g with the 15% ferrofluid soft magnet (Figure S1b, Table S1). Generally, the classification accuracies were higher when the sensor was manufactured with 15% ferrofluid than when the sensor was manufactured with 10% ferrofluid. The highest accuracies were 98.39% ± 0.93% with the RF and 98.04% ± 1.37% with the ANN when the sensor was manufactured with 15% ferrofluid, while the highest accuracy was 96.18% ± 1.41% for the ANN when the sensor was manufactured with 10% ferrofluid (Table S1).

When detecting different loads at a given taxel, the three-factor ANOVA indicated that the classification accuracies were significantly impacted by the taxel locations, soft magnets (10% ferrofluid, and 15% ferrofluid), and different algorithms (*p* < 0.01). The interactions between the machine learning algorithms and the taxel locations were significant (*p* < 0.01). One case illustrating this interaction is that the RF accuracy was significantly lower than the ANN accuracy at taxel 4 while the RF accuracy was significantly higher than the ANN accuracy at taxel 7 with the 10% ferrofluid soft magnet (Figure S1a). The interaction between locations and different ferrofluid percentage is significant (*p* < 0.01) due to the different cases like taxel 5 with 10% ferrofluid (Figure S1a) in comparison to taxel 4 with 15% ferrofluid (Figure S1b). The interaction between the four machine learning algorithms with the two different soft magnets was significant also (*p* < 0.01) due to the mean response for KNN in 10% ferrofluid being significantly different than RF in 15% ferrofluid, for example (Table S1). The three-factor interaction among load amplitude, soft magnet, and classification algorithm were also significant (*p* < 0.01) because of the significantly different cases such as the RF with 5 g load and the 10% ferrofluid soft magnet compared to the ANN with the 10 g and 15% ferrofluid soft magnet.

### 3.3. Intervertebral Monitoring of Human Spine Robotic Replica for Posture Classification

**Figure 5:**
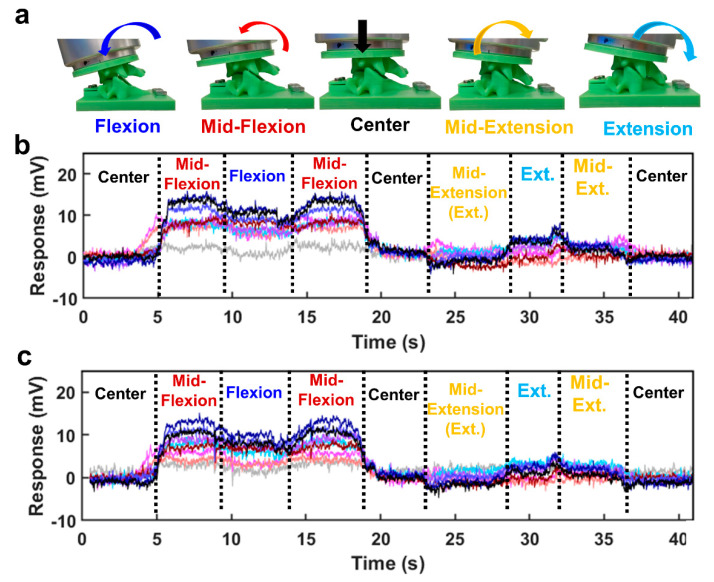
Human spine robotic replica posture measurement via intervertebral monitoring. (**a**) Five robotically actuated postures of the artificial disc-implanted spine replica were classified by four machine learning algorithms during flexion and extension motions. (**b**) Taxel responses measured with the 10% ferrofluid soft magnet. (**c**) Taxel responses measured with the 15% ferrofluid soft magnet.

Five spine postures (center, mid-flexion, flexion, mid-extension, and extension) were classified by the four machine learning algorithms with both the 10% and 15% ferrofluid soft magnetic sensor arrays. When the UR5 arm robotically flexed and extended the human spine robotic replica with the artificial disc implant (Figure 5a), the sensor signals increased or decreased significantly with both the 10% ferrofluid (Figure 5b) and 15% ferrofluid soft magnetic sensor arrays (Figure 5c). The ANN performed the best with the 10% ferrofluid soft magnetic sensor array with a 100% ± 0.0% success rate (Table 4), while both KNN and ANN performed exceptionally with the 15% magnetic sensor array with 99.66% ± 1.05% and 99.14% ± 1.81% accuracies, respectively (Table 4).

Two-factor ANOVA indicated that classification accuracies were significantly impacted (*p* < 0.01) by both the machine learning algorithms and the soft magnets. The interaction between the classification algorithms and the two soft magnets was also significant (*p* < 0.01). One example of this interaction can be seen from the mean response of the KNN, which was lower than the RF with the 10% ferrofluid soft magnet (Table 4), whereas the KNN was more accurate than the RF with the 15% ferrofluid soft magnet (Table 4).

## 4. Discussion

A new soft magnetic sensor array system was successfully fabricated to achieve intervertebral load monitoring of robotically actuated human spine replica, promising the potential to preview artificial disc implant suitability in a patient-specific fashion. Results showed that the soft magnetic sensor array system has the high capability to classify five different postures of the spine, which can be a predictor of different problems of the spine that people experience [30]. In comparing the two soft magnets on the algorithms’ abilities to detect different loads and locations, the soft magnet with 15% ferrofluid generally produced higher classification accuracy in detecting the different loads applied to each taxel (Table S1, Figure S1). As the load amplitudes increased, the soft magnets yielded very comparable classification accuracies; however, the main difference between the soft magnets was with the low amplitude loads. The location where the 10 g load was applied was classified with high accuracy using the ANN and RF algorithms with the 15% ferrofluid soft magnet (Figure 4(cii,ciii)), which was much more accurate in comparison to the 10% ferrofluid soft magnet data (Figure 3(cii)). From these findings, it is hypothesized that the sensitivity of the system could be improved by further optimizing the ratio of magnetic particles within the elastic material, which must be counterbalanced against the impact that would have upon the mechanical properties of the soft magnet sensor array. Comparing the algorithms, the ANN had classification accuracy >99% with both soft magnets during the flexion-extension experiments with the spine replica (Figure 5). However, there were several cases where the RF slightly outperformed the ANN in the probing experiments to detect the locations where the load was applied (Figure 3(cii), Figure 4(cii)). This is an important consideration to bear in mind when choosing an algorithm for a particular classification problem such as contact location, load amplitude, or more specifically, to detect the posture of the spine where the ANN performed the best.

Prior work has shown that the loads on the human cervical spine vary from 120 N to 1200 N during daily tasks [31]. However, this net load is distributed across the entire surface of the intervertebral disc and surrounding tissues. The intradiscal pressure of C3-C4 human spine cadavers ranged from 200 kPa to 270 kPa during physiologically relevant flexion and extension movements (Figure 3 in [32]). In this paper, we have applied loads ranging from 5 g to 100 g over a surface area of 4 mm^2^ with a flat-tipped probe. The 100 g load over a 4 mm^2^ area produces a physiologically relevant pressure of 245.3 kPa, which corresponds quite closely to those reported in [32]. We chose the range of loads from 5 g to 100 g to span the range from indetectable to detectable loads to uncover the capabilities of each different classification algorithm at every load and location, which was in general different. The nuanced results regarding the interplay between load amplitude and classification accuracy of the different algorithms can be helpful in the future to implement AI in new sensing applications.

The novel use of machine learning algorithms and the soft magnetic sensor array to classify different postures of a patient’s artificial disc-implanted robotic spine replica could also be utilized in conjunction with existing techniques to study and predict whether a patient is a candidate for artificial disc replacement or cage-plate fusion. Moreover, the novel system could help in determining whether a constrained, semi-constrained, or unconstrained device could be the best fit [33]. After surgery, the spine replica could assist in estimating whether there is sufficient motion at the operated level and possibly change the rehabilitation program to prevent calcification and subsequent loss of intended motion [34]. Additionally, motion and angulation data could be correlated with a patient’s symptoms or complications to better understand them from a biomechanical standpoint. In combination with wearable sensors on the patient’s spine, one could also obtain additional data to ameliorate postoperative care and the overall success of the surgery [35]. At the current time, postoperative instructions for patients with spine implants are qualitative (do as much as you can until the pain starts), creating fears in both the patient and the surgeon [36]. Questions regarding how much bending, lifting, and exercising is permissible after a cervical implant operation could be studied and correlated with biomechanical data generated by the sensorized robotic replica with individually tailored postoperative care that could be prescribed to reduce complications [37]. However, one limitation in this study is that there are no ligaments or muscles in the robotic spine replica. In the future, this could be overcome with a more lifelike tissue phantom approach or with an FEA model to understand the effect of ligaments and muscles on the sensor properties [38]. Furthermore, intervertebral simulations from the FEA model of the spine [38] could be merged with empirical measurements from the robotic replica and wearable sensors from the patient after surgeries to provide multiple viewpoints and a unified set of guidelines for post-operative care. This could lower the likelihood of complications, such as artificial disc subsidence that narrows the neuroforamen space, causing cervicalgia and cervical radiculopathy [39]. In the future, this sensor could also potentially be coupled with CT scans to address the issue of spinal malalignment [40,41,42].

## 5. Conclusions

We have created a novel robotic replica of a human spine to enable surgeons to preview the effects of surgical interventions prior to the operation. The 3D printed spine replica was modified to include an artificial disc and a soft magnetic sensor array. Benchtop experiments showed that the magnetic sensor array was readily capable of detecting the location (with 3.25 mm spacing) and amplitude of externally applied loads ≥10 g from a robotic arm, as evidenced by high classification accuracies from the four different machine learning algorithms that were compared. When the soft magnetic sensor array was integrated within the human spine robotic replica, the ANN had the highest accuracy of 100% to classify five different postures of the robotic spine replica. These results indicated that the integration of the soft magnetic sensor array within the artificial disc ‘implanted’, robotically actuated spine replica has the potential to generate physiologically relevant data before invasive surgeries, which could be used preoperatively to assess the suitability of a particular intervention for specific patients.

## Figures and Tables

**Table 1:** Statistical Features Extracted for Training Machine Learning Algorithms.

	Statistical Features for Algorithm Training
1	Mean
2	Maximum Element of a Vector
3	Minimum Element of a Vector
4	Sum of Vector Elements
5	Standard Deviation
6	Range of Values
7	Median Value
8	Root Mean Square
9	Kurtosis
10	Skewness
11	Peak Magnitude to RMS ratio
12	Most Frequent Values in an Array
13	Variance
14	Interquartile Range
15	Mean Absolute Deviation

**Table 2:** Linear fit model parameters for both soft magnets. The linear model polynomials have a form: Hall effect sensor (mV) = P1* (Load (g)) + P2.

	Soft Magnet 1 (10% Ferrofluid)			Soft Magnet 2 (15% Ferrofluid)		
Taxel	P1 (mV/g)	P2 (mV)	R^2^	P1 (mV/g)	P2 (mV)	R^2^
1	0.09	0.15	0.85	0.15	0.02	0.91
2	0.11	−0.92	0.91	0.17	0.31	0.92
3	0.07	0.18	0.87	0.12	−0.16	0.87
4	0.11	−1.45	0.92	0.18	0.86	0.94
5	0.11	−1.36	0.89	0.15	−3.12	0.91
6	0.07	−1.83	0.87	0.13	0.24	0.91
7	0.09	−1.66	0.90	0.11	−0.86	0.88
8	0.08	−1.03	0.86	0.16	0.02	0.93
9	0.05	−0.74	0.81	0.08	−0.18	0.83
Mean	0.09	−0.96	0.88	0.14	−0.32	0.90
Standard Deviation	0.02	0.73	0.03	0.03	1.15	0.04

**Table 3:** Sensor uncertainties (2) for each taxel i from both soft magnets.

	Soft Magnet 1 (10% Ferrofluid)	Soft Magnet 2 (15% Ferrofluid)
Taxel	Measurement Uncertainty (g)	Measurement Uncertainty (g)
1	6.14	5.21
2	5.21	3.81
3	8.16	5.55
4	6.87	6.24
5	4.88	4.84
6	7.15	3.23
7	7.82	4.39
8	6.35	3.34
9	11.49	5.71

**Table 4:** Machine learning algorithm accuracies to detect the five different postures of the spine replica with the 10% ferrofluid and 15% ferrofluid soft magnets.

Classification Algorithms	Accuracy (10% Ferrofluid)	Accuracy (15% Ferrofluid)
K-Nearest Neighbors (KNN)	94.00% ± 3.78%	99.66% ± 1.05%
Support Vector Machine (SVM)	97.33% ± 3.06%	99.00% ± 1.60%
Random Forest (RF)	99.33% ± 1.40%	98.33% ± 1.75%
Artificial Neural Network (ANN)	100.00% ± 0.00%	99.14% ± 1.81%

## Data Availability

The data presented in this paper will be made available upon reasonable request.

## Supplementary Materials

The following supporting information can be downloaded at: https://www.mdpi.com/article/10.3390/s22010212/s1, Figure S1: Classification accuracies from four algorithms to detect the load magnitude at each of the nine different taxels. Thirty repetitions of six different loads (5 g, 10 g, 20 g, 50 g, 75 g, 100 g) were applied to every taxel for both soft magnets. a. 10% ferrofluid soft sensor array. b. 15% ferrofluid soft sensor array; Table S1: Overall mean and standard deviation of algorithm accuracies for load amplitude classification at each of the nine taxels; Video S1: Stretchable soft magnet, load at each taxel and intervertebral monitoring of human spine replica.
