# Supplementary material for: Robotic Replica of a Human Spine Uses Soft Magnetic Sensor Array to Forecast Intervertebral Loads and Posture after Surgery
Source: Sensors (Basel). 2021 Dec 29;22(1):212. doi: 10.3390/s22010212 (PMC8749580; doi:10.3390/s22010212)
Supplement: Supplementary file 1 [file sensors-22-00212-s001.zip › Supplemental Materials Minor Revision V3.pdf]

Supplemental Document: Robotic Replica of a Human Spine Uses Soft Magnetic Sensor Array to Forecast Intervertebral Loads and Posture After Surgery

Table S1. Overall mean and standard deviation of algorithm accuracies for load amplitude classification at each of the nine taxels.

Classification Algorithms	Accuracy (*10% ferrofluid*)	Accuracy (*15% ferrofluid*)
K-Nearest Neighbors (KNN)	79.54% ± 1.73%	87.53% ± 4.85%
Support Vector Machine (SVM)	92.31% ± 2.33%	95.81% ± 2.97%
Random Forest (RF)	95.86% ± 2.20%	98.39% ± 0.93%
Artificial Neural Network (ANN)	96.18% ± 1.41%	98.04% ± 1.37%

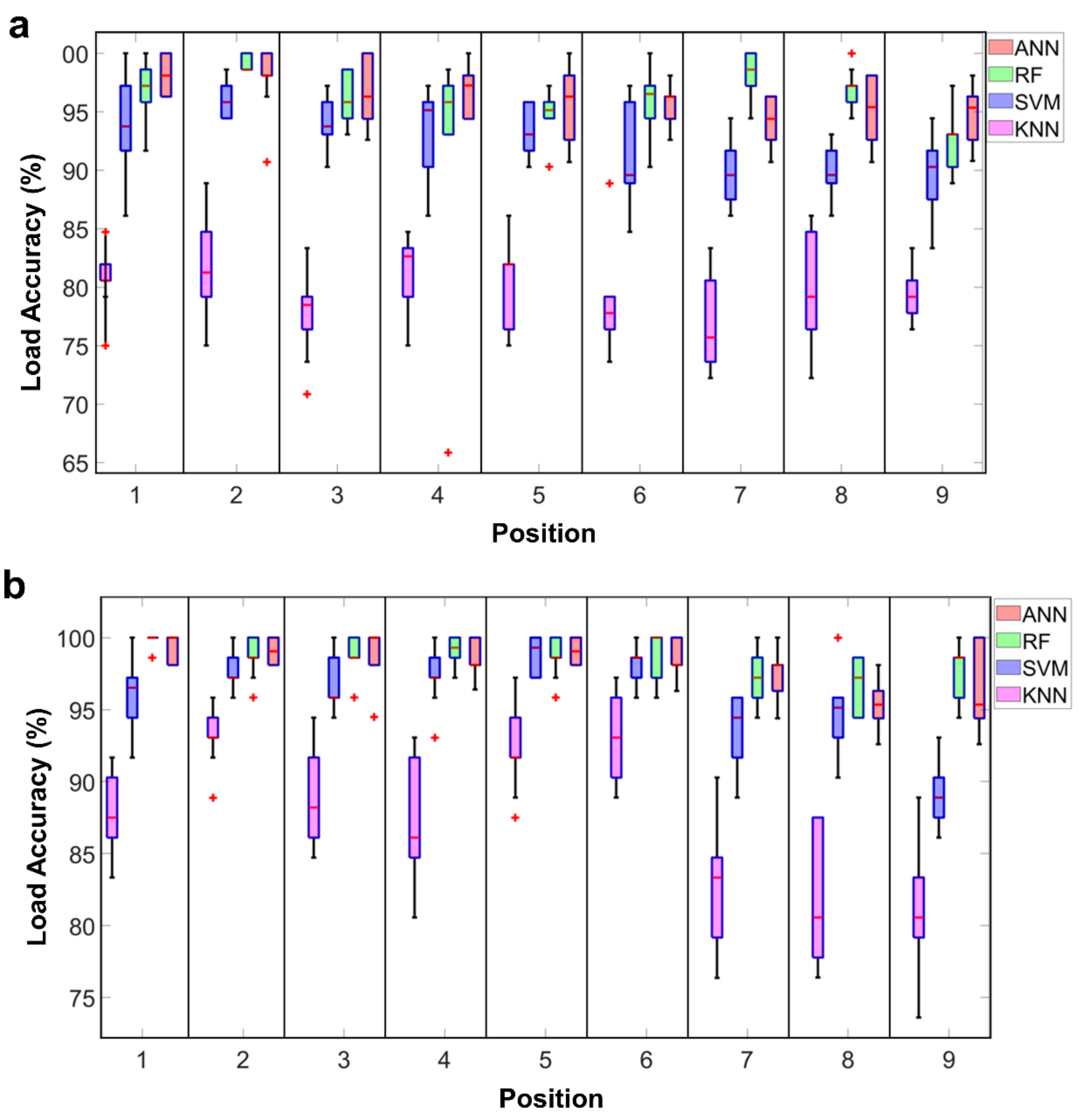

Figure S1. Classification accuracies from four algorithms to detect the load magnitude at each of the nine different taxels. Thirty repetitions of six different loads (5 g, 10 g, 20 g, 50 g, 75 g, 100 g) were applied to every taxel for both soft magnets. a. 10% ferrofluid soft sensor array. b. 15% ferrofluid soft sensor array.
